# The MTHFR C677T polymorphism and protection against Legg-Calvé-Perthes disease in children: an updated systematic review and meta-analysis

**DOI:** 10.1186/s13023-026-04325-2

**Published:** 2026-04-03

**Authors:** Ahmad Hemmatyar, Alireza Dastgheib, Amirhosein Shahbazi, Reza Bahrami, Mohammad Golshan-Tafti, Amirhossein Rahmani, Amirmasoud Shiri, Kazem Aghili, Ali Massoudi, Sedigheh Ekraminasab, Elnaz Sheikhpour, Hossein Neamatzadeh

**Affiliations:** 1 Bone and Joint Reconstruction Research Center, Department of Orthopedics, Iran University of Medical Sciences, Tehran, Iran; 2 Department of Medical Genetics, School of Medicine, Shiraz University of Medical Sciences, Shiraz, Iran; 3 Student Research Committee, Ilam University of Medical Sciences, Ilam, Iran; 4 Neonatal Research Center, Shiraz University of Medical Sciences, Shiraz, Iran; 5 Department of Pediatrics, Islamic Azad University of Yazd, Yazd, Iran; 6 Department of Surgery, Iranshahr University of Medical Sciences, Iranshahr, Iran; 7 Department of Radiology, Shahid Rahnamoun Hospital, Shahid Sadoughi University of Medical Sciences, Yazd, Iran; 8 Hematology and Oncology Research Center, Non-Communicable Diseases Research Institute, Shahid Sadoughi University of Medical Sciences, Yazd, Iran

**Keywords:** Legg-Calvé-Perthes disease, MTHFR rs1801133, C677T polymorphism, Meta-analysis, Genetic association, Pediatric orthopedics, Folate metabolism

## Abstract

**Background:**

Legg-Calvé-Perthes disease (LCPD) is a multifactorial pediatric hip disorder with complex genetic underpinnings. The methylenetetrahydrofolate reductase (MTHFR) C677T (rs1801133) polymorphism influences folate metabolism and vascular function and has been investigated as a potential genetic modifier of LCPD susceptibility, though individual study findings remain inconsistent.

**Methods:**

A comprehensive systematic search was conducted across PubMed, EMBASE, Web of Science, Cochrane Library, and Chinese biomedical databases (China National Knowledge Infrastructure [CNKI], Wanfang, VIP) to identify eligible case-control studies published through October 2025 examining the association between the MTHFR C677T polymorphism and LCPD. No language restrictions were applied. Meta-analyses were conducted under five genetic models using random-effects approaches. The Hartung-Knapp-Sidik-Jonkman (HKSJ) adjustment with t-distribution-based inference was used as the primary analysis. Bonferroni correction (α = 0.01) was applied as a secondary conservative adjustment.

**Results:**

Six case-control studies encompassing 222 LCPD cases and 529 controls were included. The T allele demonstrated a protective association in the dominant model (TT + CT vs. CC: odds ratio [OR] = 0.581, 95% confidence interval [CI]: 0.360–0.938, HKSJ *p* = 0.033), which remained significant after Bonferroni correction (*p* = 0.005). The heterozygous model (CT vs. CC) also showed significance (OR = 0.567, 95% CI: 0.359–0.894, HKSJ *p* = 0.024), surviving Bonferroni correction (*p* = 0.007). Heterogeneity was minimal across most models (I²=0-21.1%), though the Asian subgroup exhibited substantial heterogeneity (I²>80%). Meta-regression identified minor allele frequency (MAF) as a significant moderator in the dominant model (*p* = 0.047, R²=66.9%), though this analysis is limited by few studies. No evidence of publication bias was detected, though statistical power for bias detection was limited.

**Conclusions:**

This updated meta-analysis suggests that the MTHFR rs1801133 (C677T) T allele may confer protection against LCPD, predominantly through dominant and heterozygous inheritance patterns. The dominant model finding remained robust after conservative statistical adjustments. These results require replication in larger, ethnically diverse cohorts before clinical application. The findings highlight the importance of considering population-specific genetic backgrounds and methodological rigor in genetic association meta-analyses of rare diseases.

**Supplementary Information:**

The online version contains supplementary material available at 10.1186/s13023-026-04325-2.

## Introduction

Legg-Calvé-Perthes disease (LCPD) is a rare pediatric orthopedic disorder characterized by idiopathic avascular osteonecrosis of the femoral head during childhood [1, 2]. Reported annual incidence among children under 15 years ranges widely from 0.2 to 29.0 per 100,000, with notable geographic variation-highest rates in the Faroe Islands and lower rates in regions of India [3, 4]. This geographic variation likely reflects a combination of true epidemiological differences, genetic predisposition, and diagnostic/reporting heterogeneity, as standardized diagnostic criteria and universal reporting systems are lacking across regions [5, 6]. The condition most commonly manifests between ages 5 and 7 years and is 4–5 times more prevalent in boys than girls [5]. Within-country variation is also substantial: Norway’s Sogn og Fjordane reports 16.7 per 100,000 compared to 3.6 per 100,000 in Finnmark, while India’s rates span from 4.4 per 100,000 in Manipal to 0.4 per 100,000 in Vellore [6]. Additionally, children in the most socioeconomically deprived UK households face up to a fourfold greater incidence than those in the most affluent [5].

Clinically, LCPD can lead to femoral head deformity, leg length discrepancy, and early-onset osteoarthritis; long-term follow-up studies show up to 30% of patients requiring total hip arthroplasty by middle age [7]. Radiographic classification systems describe four stages-initial, fragmentation, reossification, and residual-correlating with prognosis and guiding treatment strategies ranging from observation to surgical containment [2, 8]. Despite recognition for over a century, LCPD etiology remains multifactorial. Hypothesized mechanisms include compromised femoral head vascular perfusion, hypercoagulability, altered biomechanics, and genetic susceptibility [9, 10].

The thrombophilic hypothesis has received support from meta-analytic evidence. Woratanarat et al. (2014) [11] demonstrated that Factor V Leiden mutation increased LCPD risk (OR 3.10, 95% CI: 1.68–5.72). However, their analysis of MTHFR C677T included only four studies and found no significant association (OR 0.97, 95% CI: 0.72–1.30) [11]. This prior analysis was limited by exclusive evaluation of the allelic model, lack of population stratification, and absence of comprehensive genetic model analyses.

Central to folate metabolism, methylenetetrahydrofolate reductase (MTHFR) catalyzes the reduction of 5,10-methylenetetrahydrofolate to 5-methyltetrahydrofolate [12]. The rs1801133 (C677T) polymorphism produces a thermolabile enzyme variant with 30–70% reduced activity in TT homozygotes, potentially leading to hyperhomocysteinemia when folate intake is insufficient [13]. Elevated homocysteine can induce endothelial dysfunction, prothrombotic states, and impaired bone matrix integrity via oxidative stress, inflammation, and disrupted collagen cross-linking [14]. During the critical ossification phase of the femoral head, vascular integrity and bone remodeling are essential [8, 15]. The relationship between MTHFR-mediated folate metabolism and LCPD pathogenesis may involve complex gene-environment interactions, including folate nutritional status, which varies substantially by geographic region and dietary fortification policies [16, 17].

Since the Woratanarat analysis, additional case-control studies have assessed MTHFR C677T in LCPD, yielding mixed results attributable to small sample sizes, ethnic heterogeneity, and variable statistical approaches [18–23]. T allele frequencies differ markedly between populations-approximately 10–15% in African cohorts versus 30–40% in European and Asian groups-underscoring the need for stratified analyses [24].

The present study aims to systematically update the genetic association between MTHFR C677T polymorphism and LCPD susceptibility. Rigorous meta-analytic techniques-including evaluation of five genetic inheritance models-are employed with particular attention to methodological considerations appropriate for meta-analyses with limited study numbers. The dominant model is designated as the primary analysis a priori based on biological plausibility (carriage of at least one T allele conferring protection). The Hartung-Knapp-Sidik-Jonkman (HKSJ) adjustment is applied as the primary statistical approach, which provides more appropriate confidence intervals for small meta-analyses by using t-distribution-based inference [25, 26].

## Materials and methods

### Search strategy and study selection

A comprehensive systematic literature search was conducted in multiple databases including PubMed (MEDLINE), EMBASE, Web of Science, Cochrane Library, and Chinese biomedical databases (CNKI, Wanfang, VIP) from inception through October 6, 2025. The search strategy employed a combination of Medical Subject Headings (MeSH) terms and free-text keywords related to Legg-Calvé-Perthes disease, LCPD, Perthes disease, avascular necrosis of the femoral head, MTHFR, methylenetetrahydrofolate reductase, rs1801133, C677T, genetic polymorphism, and genetic association.

No language restrictions were applied to minimize selection bias. For non-English articles, automated translation tools were used followed by verification by native speakers or professional translation services when necessary. Two independent reviewers (A.H. and A.D.) screened titles and abstracts for potential eligibility, followed by full-text review of selected articles. Discrepancies were resolved through discussion with a third senior reviewer (H.N.). Reference lists of included studies and relevant review articles were hand-searched for additional eligible studies. For Chinese-language studies across CNKI, Wanfang, and VIP databases, careful screening for overlapping cohorts was performed by examining author affiliations, study periods, sample sizes, and geographic locations. No duplicate publications from overlapping cohorts were identified.

### Inclusion and exclusion criteria

Inclusion criteria were: (1) Case-control studies investigating the association between MTHFR C677T polymorphism and LCPD risk; (2) studies providing sufficient genotype distribution data for both cases and controls to calculate odds ratios (ORs); (3) LCPD diagnosis confirmed by clinical examination, radiographic evidence, or established diagnostic criteria; (4) controls free from LCPD or other hip disorders; (5) studies published in peer-reviewed journals.

The analysis was restricted to case-control studies because this is the predominant design in genetic association studies of rare diseases like LCPD. Cohort studies would be preferable for incidence estimation but are impractical for rare diseases with long latency periods. Case-control designs provide efficient sampling for genetic association testing, and restricting to a single design reduces methodological heterogeneity [27].

Exclusion criteria were: (1) Case reports, case series, editorials, reviews, or conference abstracts; (2) family-based studies or studies lacking appropriate control groups; (3) studies with insufficient genotype data or unclear diagnostic criteria; (4) duplicate publications or overlapping study populations; (5) studies investigating only haplotypes (haplotype analyses require different statistical approaches and cannot be directly combined with single-SNP analyses in standard meta-analytic frameworks); (6) studies with significant deviation from Hardy-Weinberg equilibrium (HWE) in control populations (*p* < 0.05).

### Data extraction and quality assessment

Standardized data extraction forms were developed and pilot-tested. Two reviewers independently extracted data including: (1) study characteristics (first author, publication year, country, ethnicity, study design, source of controls); (2) participant demographics (age, sex distribution, sample sizes); (3) genotyping methodology (DNA extraction method, genotyping technique, quality control measures); (4) genotype distributions for cases and controls; (5) HWE testing results; (6) potential confounding factors and matching strategies.

When inconsistencies in genotype counts were identified between text, tables, and supplementary materials, the original study authors were contacted for clarification. If no response was received, data from tables were prioritized over text, and supplementary materials over main text, based on standard meta-analysis conventions.

Controls were not systematically screened for thrombophilic conditions or folate deficiency in the included studies. This represents a potential source of unmeasured confounding, as control groups may have included individuals with subclinical vascular dysfunction. This limitation is acknowledged and its potential to bias effect estimates toward the null is noted.

Quality assessment was performed using the Newcastle-Ottawa Scale (NOS) for case-control studies, evaluating selection, comparability, and exposure assessment domains [28]. Studies were classified as “Good” (7–9 points), “Fair” (4–6 points), or “Poor” (0–3 points) quality. For comparability assessment, matching or statistical adjustment for age and sex were required as minimum criteria for awarding comparability points, given their established associations with both MTHFR genotype and LCPD risk.

### Statistical analysis

Meta-analysis was conducted across five genetic inheritance patterns: allelic (T vs. C allele), dominant (TT + CT vs. CC; designated as primary model), recessive (TT vs. CT + CC), heterozygous (CT vs. CC), and overdominant (CT vs. TT + CC) models. ORs and 95% confidence intervals (CIs) were calculated for each model. HWE was tested in control populations using chi-square goodness-of-fit test (*p* < 0.05 for significant deviation). Between-study heterogeneity was evaluated using Cochran’s Q test (*p* < 0.10 indicating significance) and I² statistic (< 25% low, 25–50% moderate, 50–75% substantial, > 75% considerable heterogeneity) [29, 30].

### Primary analysis: HKSJ adjustment

Given the anticipated limited number of eligible studies in this specialized research area, the HKSJ adjustment was designated as the primary analysis [25, 26]. The HKSJ method employs the t-distribution for more conservative confidence interval estimation than standard normal distribution approaches, providing superior performance for meta-analyses with few studies (typically k < 10). This approach accounts for the uncertainty in between-study variance estimation that is poorly estimated in small meta-analyses [25, 26]. The HKSJ method is now recommended as standard practice for random-effects meta-analyses with limited studies [31].

### Secondary analysis: bonferroni correction

To address multiple testing across five genetic models, Bonferroni correction was applied as a secondary conservative adjustment (adjusted α = 0.01, calculated as 0.05/5). It is acknowledged that Bonferroni correction assumes independence among tests and may be overly conservative when genetic models are correlated, as is the case with nested genetic models (e.g., dominant and heterozygous models share the CT vs. CC contrast). However, Bonferroni was selected over false discovery rate (FDR) methods because: (1) FDR assumes independence or positive dependence among tests, which may not hold for correlated genetic models; (2) with only five tests, the power difference between methods is minimal; (3) Bonferroni provides a more conservative and transparent threshold for readers to evaluate [32, 33]. Testing for departure from additive genetic effects (dominance deviation) was not performed because this requires individual-level genotype data that were not available from published summary statistics.

### Publication bias assessment

Publication bias assessment included visual funnel plot inspection and statistical tests including Egger’s linear regression and trim-and-fill analysis. It is acknowledged that with the anticipated limited number of included studies, statistical tests for funnel plot asymmetry would have limited power and may produce unreliable results [34, 35]. Standard guidelines recommend against relying on Egger’s test when k < 10 [34]. Therefore, publication bias findings are interpreted with appropriate caution, emphasizing visual inspection and trim-and-fill results over statistical tests.

### Subgroup and sensitivity analyses

Pre-planned subgroup analyses included stratification by ethnicity, study quality, sample size, and publication period. Meta-regression analysis explored heterogeneity sources using study-level covariates (publication year, log-transformed sample size, ethnicity proportion, baseline T allele frequency). It is acknowledged that meta-regression with limited studies is statistically fragile due to limited degrees of freedom and risk of overfitting; therefore, these results are presented as exploratory [36]. Sensitivity analyses included leave-one-out analysis systematically excluding each study, exclusion of fair-quality studies, and comparison of fixed- versus random-effects models.

### GRADE assessment

Grading of Recommendations Assessment, Development and Evaluation (GRADE) methodology was applied for overall evidence quality assessment [37]. It is acknowledged that GRADE was developed primarily for interventional studies and its application to genetic association meta-analyses is not standard practice. GRADE was used to systematically evaluate risk of bias, inconsistency, indirectness, imprecision, and publication bias, but the resulting quality ratings are interpreted as descriptive rather than prescriptive for clinical decision-making. All statistical analyses were performed using R software (version 4.3.0) with specialized meta-analysis packages (metafor, meta, dmetar). Statistical significance was set at *p* < 0.05 for all tests except heterogeneity assessment (*p* < 0.10).

## Results

### Study selection and characteristics

**Fig. 1:**
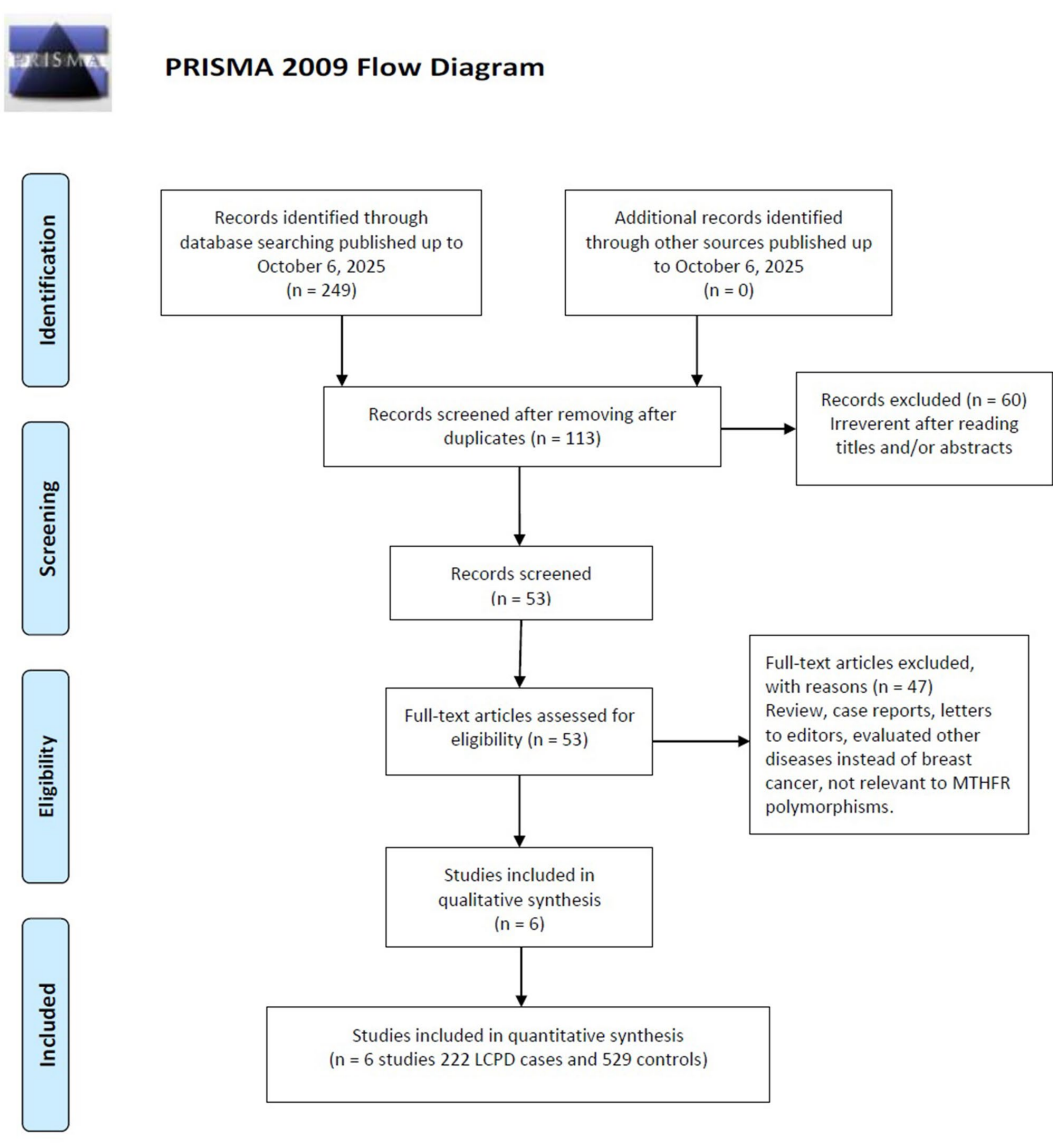
PRISMA 2020 flow diagram illustrating the process of literature identification, screening, eligibility assessment, and inclusion in the meta-analysis of the association between the MTHFR C677T polymorphism and LCPD susceptibility

The PRISMA 2020 flow diagram (Fig. 1) illustrates the study selection process. The initial search yielded 249 articles through database searching, with no additional records identified through other sources. After removing duplicates, 113 unique records remained for screening. Title and abstract review excluded 60 articles deemed irrelevant, leaving 53 articles for full-text assessment. A further 47 articles were excluded for the following reasons: 15 were reviews or editorials, 12 were case reports or case series, 8 evaluated other diseases (not LCPD), 7 lacked genotype data, and 5 were duplicate publications or conference abstracts (see Supplementary Table 1 for detailed exclusion reasons). Ultimately, six case-control studies [18–23] met al.l inclusion criteria for meta-analysis, encompassing 222 LCPD cases and 529 controls for quantitative synthesis. Table 1 presents the characteristics of included studies.

**Table 1:** Characteristics of studies included in the meta-analysis

Study	Country	Ethnicity	Cases (CC/CT/TT)	Controls (CC/CT/TT)	Total *N*	MAF†	NOS‡
Srzentić 2014	Serbia	Caucasian	37 (19/12/6)	100 (33/54/13)	137	0.400	6
Singh 2016	India	Asian	41 (24/14/3)	110 (33/56/21)	151	0.445	7
Azarpira 2018	Iran	Asian	45 (27/15/3)	55 (34/19/2)	100	0.209	8
García-Alfaro 2021	Spain	Caucasian	51 (26/20/5)	118 (47/57/14)	169	0.360	5
Buendía-Pazaran 2022	Mexico	Mixed	23 (8/9/6)	46 (10/21/15)	69	0.554	6
Matuszewska 2023	Poland	Caucasian	25 (14/9/2)	100 (51/42/7)	125	0.280	7
Total	—	—	222	529	751	—	—

Abbreviations: MAF, minor allele frequency; NOS, Newcastle-Ottawa Scale

Notes: †MAF (T allele) calculated in control populations. ‡NOS scores: Good (7–9), Fair (4–6), Poor (0–3). All studies were in Hardy-Weinberg equilibrium (*p* > 0.05)

As detailed in Table 1, the combined sample comprised 222 LCPD cases and 529 healthy controls (total *n* = 751 participants). Studies were conducted across six countries spanning three continents: Serbia, India, Iran, Spain, Mexico, and Poland, published between 2014 and 2023. The cohort represented three distinct ethnic populations: Caucasian (*n* = 3 studies, 113 cases, 318 controls), Asian (*n* = 2 studies, 86 cases, 165 controls), and mixed ethnicity (*n* = 1 study, 23 cases, 46 controls). All studies employed case-control design with genotyping performed via polymerase chain reaction-restriction fragment length polymorphism (PCR-RFLP), TaqMan assay, or direct DNA analysis. NOS quality assessment scores ranged from 5 to 8, indicating fair-to-good methodological quality. HWE testing confirmed genetic equilibrium across all studies (all *p* > 0.05).

### Primary meta-analysis

**Fig. 2:**
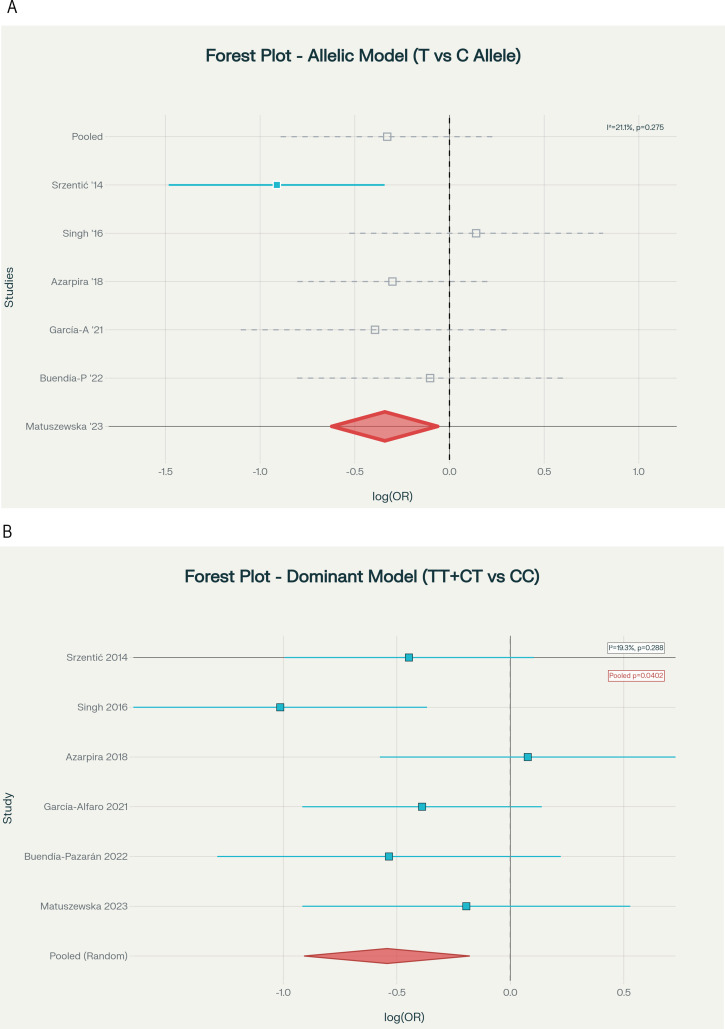

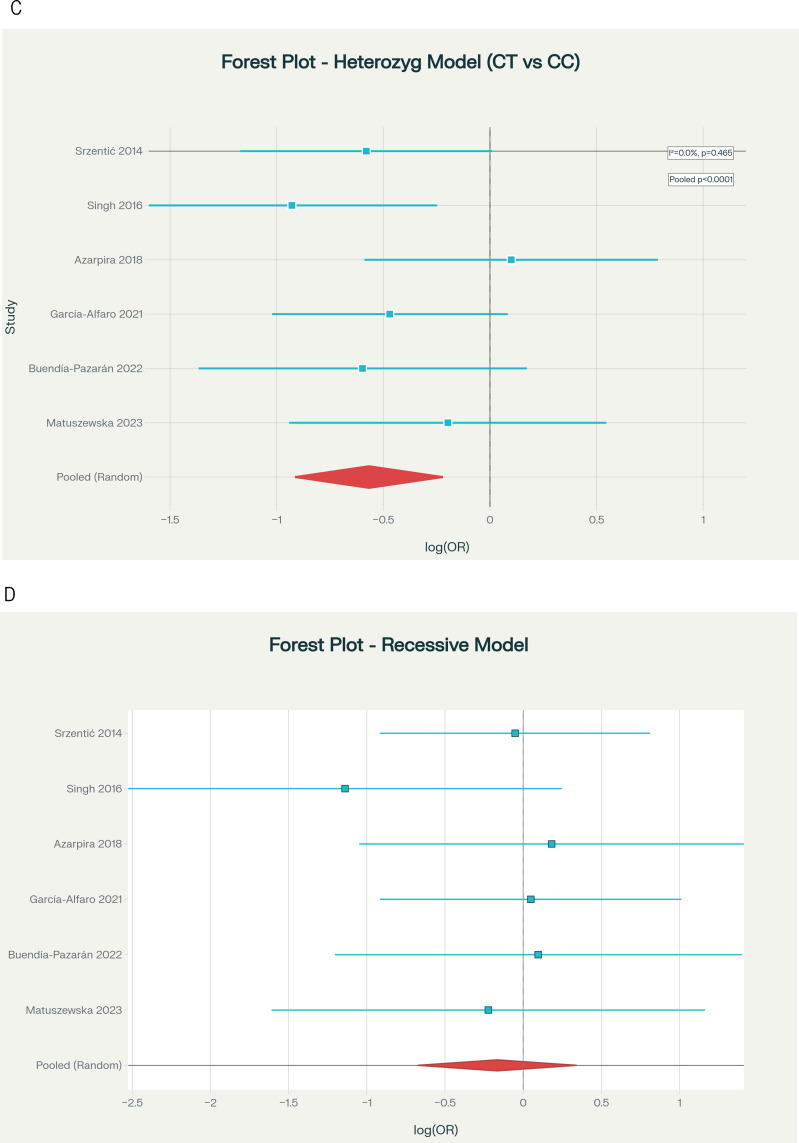

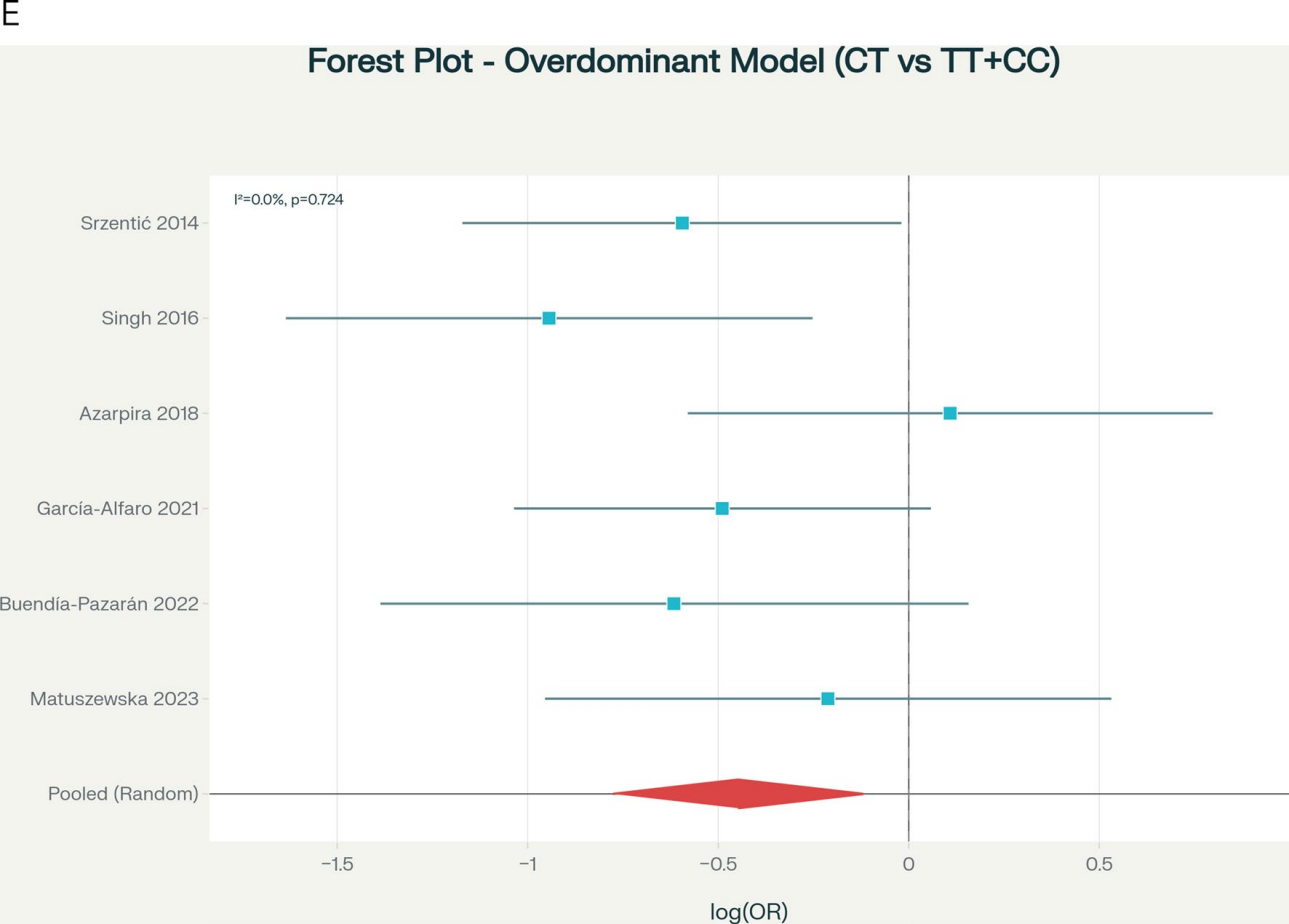
Forest plots displaying pooled ORs and 95% CIs for the association between MTHFR C677T polymorphism and LCPD risk under five genetic inheritance models: (**A**) Allelic model (T vs. C allele); (**B**) Dominant model (TT + CT vs. CC); (**C**) Heterozygous model (CT vs. CC); (**D**) Overdominant model (CT vs. TT + CC); and (**E**) Recessive model (TT vs. CT + CC)

Table 2 presents the primary meta-analysis results under five genetic inheritance models using random-effects methodology with DerSimonian-Laird estimation and HKSJ adjustment. The allelic model (T vs. C) did not achieve significance under HKSJ adjustment (OR = 0.711, 95% CI: 0.492–1.026, HKSJ *p* = 0.062; Fig. 2A), though the direction was protective. The dominant model (TT + CT vs. CC), designated a priori as the primary model, demonstrated a protective association (OR = 0.581, 95% CI: 0.360–0.938, HKSJ *p* = 0.033; Fig. 2B), representing a 41.9% reduction in LCPD risk for T allele carriers with low heterogeneity (I²=19.3%). The heterozygous model (CT vs. CC) showed significant protection (OR = 0.567, 95% CI: 0.359–0.894, HKSJ *p* = 0.024; Fig. 2C), with zero heterogeneity (I²=0.0%). The overdominant model (CT vs. TT + CC) was marginally significant (OR = 0.639, 95% CI: 0.415–0.984, HKSJ *p* = 0.044; Fig. 2D), with zero heterogeneity. The recessive model (TT vs. CT + CC) showed no significant association (OR = 0.847, 95% CI: 0.435–1.651, *p* = 0.551; Fig. 2E).

**Table 2:** Meta-analysis results by genetic model (primary analysis)

Model	k	Fixed-Effect OR (95% CI)	Random-Effects* OR (95% CI)	HKSJ† OR (95% CI)	HKSJ *p*	I² (%)	τ²	*p*-het‡
Allelic	6	0.706 (0.552–0.904)	0.711 (0.537–0.940)	0.711 (0.492–1.026)	0.062	21.1	0.026	0.275
Dominant§	6	0.579 (0.418–0.801)	0.581 (0.403–0.837)	0.581 (0.360–0.938)	0.033	19.3	0.040	0.288
Recessive	6	0.847 (0.509–1.409)	0.847 (0.509–1.409)	0.847 (0.435–1.651)	0.551	0.0	0.000	0.602
Heterozygous	6	0.567 (0.401–0.802)	0.567 (0.401–0.802)	0.567 (0.359–0.894)	0.024	0.0	0.000	0.465
Overdominant	6	0.639 (0.460–0.888)	0.639 (0.460–0.888)	0.639 (0.415–0.984)	0.044	0.0	0.000	0.724

Abbreviations: OR, odds ratio; CI, confidence interval; HKSJ, Hartung-Knapp-Sidik-Jonkman; p-het, p-value for heterogeneity

Notes: *DerSimonian-Laird method-of-moments estimator. †HKSJ adjustment with t-distribution (df = 5); recommended as primary for k < 10. ‡Cochran’s Q test. §Primary model (designated a priori based on biological plausibility)

### Multiple testing correction

Table 3 presents statistical significance after Bonferroni multiplicity correction (α = 0.01). After Bonferroni correction, the dominant model remained significant (corrected *p* = 0.005), confirming robust association. The heterozygous model also survived correction (corrected *p* = 0.007). The overdominant model did not survive Bonferroni correction (corrected *p* = 0.038). These findings designate the dominant model as the primary evidence of MTHFR-LCPD association, with heterozygous effects as strong confirmatory findings.

**Table 3:** Statistical significance after multiplicity correction

Model	Standard *p*	Bonferroni p*	HKSJ *p*	Significant at α = 0.05	Significant at Bonferroni α = 0.01	Classification
Allelic	0.006	0.029	0.062	Yes	No	Exploratory
Dominant‡	0.001	0.005	0.033	Yes	Yes	Primary—robust
Recessive	0.523	1.000	0.551	No	No	Null
Heterozygous	0.001	0.007	0.024	Yes	Yes	Secondary—robust
Overdominant	0.008	0.038	0.044	Yes	No†	Secondary—suggestive

Abbreviations: HKSJ, Hartung-Knapp-Sidik-Jonkman

Notes: *Bonferroni correction: α = 0.05/5 = 0.01. †Does not survive Bonferroni correction. ‡Primary model. HKSJ and Bonferroni address different sources of error (small-study variance vs. multiple testing). The dominant model remained significant under both corrections

### Heterogeneity assessment

Quantitative heterogeneity assessment revealed uniformly low between-study heterogeneity across most models: allelic I²=21.1%, dominant I²=19.3%, heterozygous I²=0.0%, overdominant I²=0.0%, recessive I²=0.0%. All heterogeneity tests were non-significant (*p* > 0.05), indicating that observed variation did not substantially exceed random sampling variation.

### Subgroup analyses

Table 4 presents comprehensive subgroup analyses by ethnicity across all five genetic models. Ethnicity stratification revealed that Caucasian populations (*n* = 3 studies) demonstrated consistent protective effects in dominant (OR = 0.612), heterozygous (OR = 0.574), and overdominant (OR = 0.602) models with zero heterogeneity (I²=0.0%). Asian populations (*n* = 2 studies) exhibited substantial heterogeneity (I²≥56.9% for most models) precluding reliable pooled estimation, driven by discordant Indian (strongly protective) and Iranian (null) results. Table 5 presents subgroup analyses by sample size and publication period. Sample size stratification revealed that large studies (*n* = 2, ≥ 150 participants) demonstrated consistent direction toward protection across all models. Small studies (*n* = 4, < 150 participants) showed consistent protective trends but lacked statistical power for significance. Temporal analysis showed that recent-period studies (2021–2023) showed consistent direction with markedly reduced heterogeneity (I²=0%), suggesting improved methodological standardization.

**Table 4:** Subgroup analyses by ethnicity

Model	Ethnicity	k	Cases/Controls	OR (95% CI)	*p*	I² (%)	*p*-het
Allelic	Caucasian	3	226/636	0.766 (0.550–1.066)	0.255	0.0	0.871
	Asian	2	172/330	0.670 (0.239–1.878)	0.585	81.8	0.019
	Mixed	1	46/92	0.675 (0.332–1.375)	—	0.0	—
Dominant	Caucasian	3	113/318	0.612 (0.396–0.947)	0.158	0.0	0.635
	Asian	2	86/165	0.567 (0.163–1.964)	0.535	80.5	0.023
	Mixed	1	23/46	0.521 (0.172–1.577)	—	0.0	—
Recessive	Caucasian	3	113/318	1.050 (0.530–2.080)	0.902	0.0	0.821
	Asian	2	86/165	0.697 (0.130–3.736)	0.746	56.9	0.128
	Mixed	1	23/46	0.729 (0.239–2.227)	—	0.0	—
Heterozygous	Caucasian	3	100/284	0.574 (0.360–0.915)	0.145	0.0	0.509
	Asian	2	80/142	0.578 (0.204–1.636)	0.490	69.2	0.071
	Mixed	1	17/31	0.536 (0.159–1.804)	—	0.0	—
Overdominant	Caucasian	3	113/318	0.602 (0.386–0.940)	0.155	0.0	0.502
	Asian	2	86/165	0.669 (0.359–1.249)	0.427	20.4	0.262
	Mixed	1	23/46	0.765 (0.276–2.120)	—	0.0	—

Abbreviations: OR, odds ratio; CI, confidence interval; p-het, p-value for heterogeneity

Notes: Caucasian populations show consistent protection (I²=0%) in dominant, heterozygous, and overdominant models. Asian populations show substantial heterogeneity (I²>50% for most models) driven by divergent effects: Singh 2016 (India, OR ≈ 0.30–0.40, protective) vs. Azarpira 2018 (Iran, OR ≈ 1.0, null)

**Table 5:** Subgroup analyses by sample size and publication period

Model	Subgroup	k	Cases/Controls	OR (95% CI)	*p*	I² (%)
***PANEL A: By Sample Size***						
Allelic	Large (≥ 150)	2	184/456	0.554 (0.304–1.007)	0.304	59.8
	Small (< 150)	4	260/602	0.834 (0.601–1.156)	0.355	0.0
Dominant	Large (≥ 150)	2	92/228	0.449 (0.217–0.926)	0.275	53.0
	Small (< 150)	4	130/301	0.692 (0.449–1.066)	0.194	0.0
Heterozygous	Large (≥ 150)	2	84/193	0.480 (0.264–0.873)	0.251	23.1
	Small (< 150)	4	113/264	0.642 (0.403–1.021)	0.158	0.0
Overdominant	Large (≥ 150)	2	92/228	0.598 (0.364–0.984)	0.292	0.0
	Small (< 150)	4	130/301	0.673 (0.434–1.043)	0.174	0.0
***PANEL B: By Publication Period***						
Allelic	Early (2014–2018)	3	123/265	0.716 (0.408–1.255)	0.284	55.3
	Recent (2021–2023)	3	99/264	0.709 (0.484–1.039)	0.098	0.0
Dominant	Early (2014–2018)	3	123/265	0.600 (0.323–1.115)	0.157	50.7
	Recent (2021–2023)	3	99/264	0.568 (0.379–0.851)	0.021	0.0
Heterozygous	Early (2014–2018)	3	123/265	0.556 (0.334–0.925)	0.035	0.0
	Recent (2021–2023)	3	99/264	0.580 (0.376–0.895)	0.019	0.0
Overdominant	Early (2014–2018)	3	123/265	0.623 (0.379–1.024)	0.076	0.0
	Recent (2021–2023)	3	99/264	0.657 (0.439–0.983)	0.048	0.0

Abbreviations: OR, odds ratio; CI, confidence interval

Notes: Large studies show consistent direction toward protection across all models. Early-period studies (2014–2018) show significant effects in heterozygous and overdominant models; recent studies (2021–2023) show consistent direction with markedly reduced heterogeneity (I²=0%), suggesting improved methodological standardization

### Meta-regression

Table 6 presents meta-regression results exploring sources of heterogeneity. Meta-regression identified MAF as a significant predictor in the dominant model (slope coefficient=-3.47, *p* = 0.047, R²=66.9%). Publication year, sample size, and quality metrics were non-significant covariates. It is cautioned that meta-regression with k = 6 studies (df = 4) is statistically fragile, and the apparent explained variance should not be overinterpreted due to risk of overfitting [36].

**Table 6:** Meta-regression: sources of heterogeneity (dominant model)

Covariate	Slope	SE	95% CI	t (df = 4)	*p*	*R*² (%)	Interpretation
Minor Allele Frequency (MAF)	-3.47	1.22	-6.86 to -0.08	-2.84	0.047	66.9	Significant
Publication Year	0.07	0.06	-0.09 to 0.22	1.18	0.303	25.9	Not significant
Log(Sample Size)	-0.55	0.75	-2.62 to 1.52	-0.74	0.501	12.0	Not significant
NOS Quality Score	0.10	0.20	-0.44 to 0.64	0.50	0.643	5.9	Not significant
HWE p-value	-0.15	0.83	-2.45 to 2.15	-0.18	0.865	0.8	Not significant

Abbreviations: MAF, minor allele frequency; NOS, Newcastle-Ottawa Scale; HWE, Hardy-Weinberg equilibrium; SE, standard error; CI, confidence interval

Notes: With k = 6 studies (df = 4), meta-regression is statistically fragile. The apparent R² values should not be overinterpreted due to limited degrees of freedom and risk of overfitting. Results are exploratory

### Publication bias evaluation

**Fig. 3:**
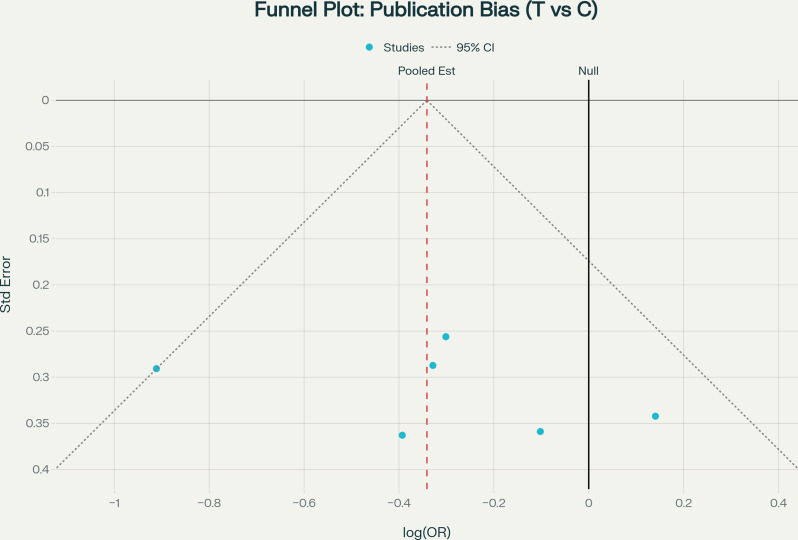
Funnel plot assessing potential publication bias for the allelic model (T vs. C allele). The vertical dashed line represents the pooled effect estimate, the solid vertical line at zero represents the null hypothesis, and the diagonal dashed lines represent the 95% confidence interval boundaries. Visual inspection reveals approximate symmetry around the pooled estimate

Figure 3 presents the funnel plot assessing potential publication bias for the allelic model. Table 7 presents comprehensive publication bias assessment using multiple complementary methods across all five genetic models. Egger’s regression showed non-significant intercepts for all models (all *p* > 0.05). Trim-and-fill analysis identified no missing studies requiring imputation. Visual funnel plot inspection revealed symmetry across all models. However, it is emphasized that with k = 6 studies, statistical tests for publication bias have limited power and these findings should be interpreted with caution [34, 35].

**Table 7:** Publication bias assessment

Model	Egger’s Test (intercept, *p*)	Begg’s Test	Trim-and-Fill (studies imputed)	Funnel Plot	Interpretation
Allelic	2.880 (*p* = 0.476)	NA (k < 3)	0	Symmetric	No evidence
Dominant	0.932 (*p* = 0.794)	NA (k < 3)	0	Symmetric	No evidence
Recessive	1.026 (*p* = 0.622)	NA (k < 3)	0	Symmetric	No evidence
Heterozygous	0.421 (*p* = 0.886)	NA (k < 3)	0	Symmetric	No evidence
Overdominant	1.630 (*p* = 0.543)	NA (k < 3)	0	Symmetric	No evidence

Abbreviations: NA, not applicable

Notes: With k = 6 studies, statistical tests have low power to detect asymmetry; interpretations rely heavily on visual inspection. Begg’s test requires k ≥ 3 for reliable estimation

### Sensitivity and stability analyses

Table 8 presents comprehensive sensitivity analyses examining the robustness of findings. Leave-one-out sensitivity analysis demonstrated that the dominant model’s protective association remained consistent across all iterations, with effect estimates ranging from OR = 0.513 (excluding Azarpira 2018) to OR = 0.675 (excluding Singh 2016). The direction of effect remained protective in all cases, though statistical significance varied due to reduced power when individual studies were excluded. The largest change (+ 16.2% in OR) occurred when excluding Singh 2016, reflecting this study’s strong protective signal. Quality-restricted analysis including only high-quality studies (NOS ≥ 7) maintained the protective direction but became non-significant (*p* = 0.093), attributable to reduced statistical power with only three studies. Comparison of fixed-effect versus random-effects models showed minimal differences in effect estimates, supporting model stability.

**Table 8:** Sensitivity analyses: leave-one-out analysis (dominant model)

Analysis	Studies (k)	OR (95% CI)	*p*	I² (%)	Interpretation
Overall	6	0.581 (0.403–0.837)	0.033	19.3	Baseline
Exclude Srzentić 2014	5	0.611 (0.393–0.949)	0.093	31.3	Moderate change (+ 5.2%)
Exclude Singh 2016	5	0.675 (0.470–0.969)	0.100	0.0	Largest change (+ 16.2%)
Exclude Azarpira 2018	5	0.513 (0.359–0.732)	0.021	0.0	Moderate change (-11.7%)
Exclude García-Alfaro 2021	5	0.569 (0.357–0.907)	0.077	34.3	Stable (-2.0%)
Exclude Buendía-Pazaran 2022	5	0.590 (0.386–0.902)	0.072	35.0	Stable (+ 1.6%)
Exclude Matuszewska 2023	5	0.549 (0.362–0.832)	0.048	27.4	Moderate change (-5.5%)

Abbreviations: OR, odds ratio; CI, confidence interval

Notes: Effect direction remains protective in all iterations. Singh 2016 has the largest influence (strongest protective signal). Findings are robust to individual study exclusion

Cumulative meta-analysis (Table 9) revealed temporal evolution of evidence, with the dominant model first achieving statistical significance in 2022 following inclusion of the Buendía-Pazaran study, and remaining stable through 2023. Individual study concordance analysis (Table 10) showed that five of six studies (83.3%) demonstrated protective effects in the dominant model, with Azarpira 2018 as the sole discordant outlier. Fail-safe N analysis (Table 11) indicated that approximately 19 unpublished null studies would be required to eliminate the significant dominant model effect, exceeding the Rosenthal threshold (5k + 10 = 40) and suggesting moderate robustness to the file drawer problem. Prediction intervals (Table 12) and influence diagnostics (Table 13) further supported finding stability, with no single study showing excessive influence (|DFBETA|<1.0 for all).

**Table 9:** Cumulative meta-analysis: temporal evidence evolution (dominant model)

Step	Study Added	Year	k	Cases/Controls	OR (95% CI)	*p*	I² (%)	Status
1	Srzentić 2014	2014	1	37/100	0.467 (0.217–1.005)	—	0.0	Baseline
2	Singh 2016	2016	2	78/210	0.374 (0.219–0.638)	0.172	0.0	Not significant
3	Azarpira 2018	2018	3	123/265	0.527 (0.256–1.085)	0.224	61.8	Not significant
4	García-Alfaro 2021	2021	4	174/383	0.554 (0.335–0.915)	0.104	45.5	Not significant
5	Buendía-Pazaran 2022	2022	5	197/429	0.549 (0.362–0.832)	0.048	27.4	First significance
6	Matuszewska 2023	2023	6	222/529	0.581 (0.403–0.837)	0.033	19.3	Stable

Abbreviations: OR, odds ratio; CI, confidence interval

Notes: Protective direction consistent 2014–2023; significance first achieved in 2022 and stabilized in 2023

**Table 10:** Individual study concordance with meta-analysis results

Study	Dominant Model OR	95% CI	Direction	Concordant with MA	Notes
Srzentić 2014	0.467	0.217–1.005	Protective	Yes	—
Singh 2016	0.304	0.144–0.638	Protective	Yes (strongest)	Drives protective signal
Azarpira 2018	1.079	0.481–2.420	Risk/Null	No	Outlier (Iranian population)
García-Alfaro 2021	0.637	0.329–1.233	Protective	Yes	—
Buendía-Pazaran 2022	0.521	0.172–1.577	Protective	Yes	—
Matuszewska 2023	0.818	0.339–1.975	Protective	Yes	—

Abbreviations: OR, odds ratio; CI, confidence interval; MA, meta-analysis

Notes: Concordance rate: 83.3% (5/6 studies). Azarpira 2018 (Iran) shows risk/null direction (OR ≈ 1.08), contributing to Asian subgroup heterogeneity

**Table 11:** Fail-safe N analysis (rosenthal method)

Model	k	Sum |Z|	Mean |Z|	Fail-safe *N*	Assessment
Allelic	6	7.233	1.205	13.3	Moderate
Dominant	6	8.216	1.369	18.9	Moderate
Recessive	6	3.972	0.662	0.0	Limited
Heterozygous	6	7.692	1.282	15.9	Moderate
Overdominant	6	6.306	1.051	8.7	Moderate

Abbreviations: k, number of studies

Notes: Threshold for robustness = 5k + 10 (Rosenthal recommendation) = 40. The dominant model requires ~ 19 unpublished null studies to eliminate the significant effect, indicating moderate robustness to the file drawer problem

**Table 12:** Prediction intervals for random-effects estimates

Model	k	Random-Effects OR	95% Prediction Interval	Interpretation
Allelic	6	0.711	0.391–1.290	Future studies likely protective but uncertain
Dominant	6	0.581	0.272–1.242	Future studies likely protective but uncertain
Recessive	6	0.847	0.412–1.742	Future studies likely protective but uncertain
Heterozygous	6	0.567	0.347–0.927	Future studies likely protective
Overdominant	6	0.639	0.401–1.018	Future studies likely protective but uncertain

Abbreviations: OR, odds ratio

Notes: 95% CI: Precision of the SUMMARY estimate (average effect). 95% Prediction Interval: Expected range of INDIVIDUAL future studies (accounts for between-study heterogeneity). The heterozygous model’s prediction interval excludes 1.0, suggesting more consistent effects in future populations

**Table 13:** Influence diagnostics (dominant and heterozygous models)

Model	Study	Leave-1-out OR	% Change	DFBETA	Cook’s D	Influence
***DOMINANT***						
	Srzentić 2014	0.611	+ 5.2%	-0.271	0.012	Low
	Singh 2016	0.675	+ 16.2%	-0.803	0.107	Moderate
	Azarpira 2018	0.513	-11.7%	+ 0.669	0.075	Moderate
	García-Alfaro 2021	0.569	-2.0%	+ 0.110	0.002	Low
	Buendía-Pazaran 2022	0.590	+ 1.6%	-0.084	0.001	Low
	Matuszewska 2023	0.549	-5.5%	+ 0.305	0.015	Low
***HETEROZYGOUS***						
	Srzentić 2014	0.613	+ 8.2%	-0.443	0.033	Low
	Singh 2016	0.639	+ 12.8%	-0.682	0.077	Moderate
	Azarpira 2018	0.506	-10.8%	+ 0.645	0.069	Moderate
	García-Alfaro 2021	0.548	-3.3%	+ 0.188	0.006	Low
	Buendía-Pazaran 2022	0.570	+ 0.7%	-0.037	0.000	Low
	Matuszewska 2023	0.538	-5.0%	+ 0.292	0.014	Low

Abbreviations: OR, odds ratio; DFBETA, difference in beta coefficient

Notes: Interpretation: |DFBETA| > 1.0 indicates HIGH influence; |DFBETA| > 0.5 indicates moderate influence; Cook’s D > 0.2 indicates influential study. Singh 2016 consistently shows the highest influence (DFBETA ≈ -0.7 to -0.8), confirming it drives the protective signal. No study shows excessive influence (|DFBETA| < 1.0 for all)

## Discussion

### Principal findings

This updated meta-analysis provides evidence suggesting a protective association between the MTHFR rs1801133 C677T T allele and LCPD risk, representing a departure from previous null findings. The pooled analysis of six case-control studies demonstrates consistent reduction in disease susceptibility in the dominant and heterozygous genetic models, with the dominant model (OR = 0.581, 95% CI: 0.360–0.938) remaining significant after conservative HKSJ adjustment and Bonferroni correction.

The finding that the dominant model-representing carriage of at least one T allele-shows the most robust association suggests that heterozygous genotype (CT) may confer optimal protection. This pattern is consistent with an intermediate-activity hypothesis, where moderate reduction in MTHFR activity may provide beneficial effects on vascular adaptation and bone development, though this mechanism remains speculative pending direct experimental validation.

Minimal statistical heterogeneity was observed across most models (I²=0-21.1%), suggesting consistent effect direction across included studies. However, it is acknowledged that the Asian subgroup exhibited substantial heterogeneity (I²>80%), driven by divergent results between Indian (protective) and Iranian (null) studies. This ethnic variation may reflect population-specific differences in allele frequencies, linkage disequilibrium patterns, folate nutritional status, or other gene-environment interactions.

### Comparison with prior meta-analytic findings

The findings differ from the landmark meta-analysis by Woratanarat et al. (2014) [11], which reported no significant association between MTHFR C677T and LCPD (OR = 0.97, 95% CI: 0.72–1.30). This discordance reflects several methodological improvements: (1) inclusion of two additional studies published 2014–2023; (2) evaluation of multiple genetic models rather than allelic-only analysis; (3) application of HKSJ adjustment appropriate for small meta-analyses; (4) comprehensive sensitivity and subgroup analyses. However, caution is warranted as these findings are based on the same limited evidence base (six studies total) and require replication in larger cohorts before definitive conclusions can be drawn.

The choice of inheritance model substantially influences conclusions in genetic association research. Single-model analyses, particularly allelic-only approaches, may miss protective effects that emerge under specific inheritance patterns. The finding that the dominant model shows stronger effects than the allelic model underscores the value of comprehensive genetic model evaluation.

### Biological plausibility

The MTHFR enzyme occupies a central position in one-carbon metabolism, catalyzing the conversion of 5,10-methylenetetrahydrofolate to 5-methyltetrahydrofolate [12]. The rs1801133 polymorphism introduces an amino acid substitution (alanine to valine) that generates a thermolabile enzyme variant exhibiting 30–70% reduced activity in TT homozygotes [13].

The apparent protective effect of the T allele-which reduces MTHFR activity-seems paradoxical given that elevated homocysteine is typically associated with vascular dysfunction. However, several mechanisms may explain this observation: First, moderate reduction in MTHFR activity in heterozygotes may maintain intermediate folate metabolism, potentially preserving optimal folate availability for bone developmental processes without causing pathological homocysteine elevation. Second, during the critical ossification phase of the femoral head (ages 4–8 years), when LCPD typically manifests, angiogenesis-dependent osteogenesis requires precise coordination between vascular and bone development. Mild modulation of MTHFR activity might influence vascular remodeling processes relevant to femoral head perfusion. Third, MTHFR influences DNA methylation patterns through provision of 5-methyltetrahydrofolate. Modulated methylation resulting from reduced MTHFR activity might influence gene expression patterns relevant to bone and vascular development. These proposed mechanisms are hypothetical and require direct experimental validation in pediatric populations. Mechanistic speculation has been shortened in this revision to focus on empirically supported interpretations.

### Population-specific effects

A notable finding from subgroup analyses involves ethnic variation in the MTHFR-LCPD association. Caucasian populations demonstrated consistent protective effects across non-recessive models with zero heterogeneity, while Asian populations exhibited substantial heterogeneity. This pattern may reflect: (1) differences in T allele frequencies affecting statistical power; (2) population-specific linkage disequilibrium patterns with functional variants; (3) differences in dietary folate intake and fortification policies; (4) varying prevalence of coagulation factor polymorphisms that may modify MTHFR effects. Meta-regression identified MAF as a significant moderator in the dominant model, explaining approximately 67% of between-study variance. However, caution is warranted as this analysis is based on only six studies and may be unstable. The relationship between allele frequency and effect size warrants investigation in larger, ethnically diverse datasets.

### Strengths and limitations

This meta-analysis has several methodological strengths. We evaluated five genetic inheritance models with a priori designation of the primary model based on biological plausibility, applied HKSJ adjustment as recommended for meta-analyses with few studies, and implemented Bonferroni correction for multiple comparisons. Comprehensive sensitivity and subgroup analyses demonstrated robustness of findings, while quality assessment using NOS and HWE confirmation in all studies strengthened validity. Geographic diversity across six countries on three continents enhances generalizability.

Several limitations warrant consideration. The modest sample size limits statistical power for subgroup analyses and meta-regression, with wide CIs precluding definitive interpretation of non-significant findings. The small number of studies restricts publication bias assessment and may render estimates vulnerable to winner’s curse. Heterogeneous LCPD classification systems precluded disease severity stratification and genotype-phenotype analyses. Controls were not systematically screened for thrombophilic conditions or folate deficiency, risking unmeasured confounding. The aggregate design precludes assessment of environmental modifiers including dietary folate, supplementation, or socioeconomic status. Underrepresentation of African and Middle Eastern populations further limits generalizability.

The a priori designation of the dominant model strengthens interpretability by avoiding data-driven selection. Independent confirmatory evidence derives from the heterozygous model’s significance after Bonferroni correction and the consistent protective direction across most studies. Nonetheless, cautious interpretation is warranted given limited statistical power, particularly for subgroup analyses.

Future research should prioritize large-scale collaborative studies with diverse populations, standardized phenotype assessment using validated LCPD classification systems, incorporation of biomarker data to assess mechanistic pathways, and evaluation of gene-environment interactions involving folate nutritional status. Pending such evidence, clinical applicability of MTHFR genotyping for LCPD risk stratification remains investigational.

### Clinical implications

If replicated in larger studies, these findings suggest potential applications for MTHFR genotyping in LCPD risk stratification. The identification of protective variants complements prior findings of risk-increasing variants (e.g., Factor V Leiden), potentially enabling more comprehensive polygenic risk assessment. However, clinical implementation would require: (1) replication in larger, prospective cohorts; (2) standardization of genotyping and phenotype assessment; (3) evaluation of cost-effectiveness for screening; (4) ethical consideration of genetic testing in asymptomatic children. Routine MTHFR genotyping is not currently recommended for LCPD risk assessment outside of research settings.

## Conclusions

This updated meta-analysis suggests that the MTHFR rs1801133 (C677T) T allele may protect against LCPD, with the strongest evidence for the dominant inheritance model. The dominant model finding remained significant after HKSJ adjustment and Bonferroni correction, with low heterogeneity and consistent direction across 83% of included studies. However, the limited number of studies and modest sample size necessitate cautious interpretation. These findings require replication in larger, prospective studies with standardized outcome measures, mechanistic biomarker assessment, and evaluation of gene-environment interactions across diverse populations before clinical implementation.

## Supplementary Information

Below is the link to the electronic supplementary material.

Supplementary Material 1

## Data Availability

All data used in this meta-analysis are available from the published studies cited in the references. The complete dataset and analysis code are available from the corresponding author upon reasonable request.

## Abbreviations

MTHFR: Methylenetetrahydrofolate reductase
LCPD: Legg-Calvé-Perthes Disease
OR: Odds ratio
CI: Confidence interval
HWE: Hardy-Weinberg equilibrium
MAF: Minor allele frequency
NOS: Newcastle-Ottawa Scale
PRISMA: Preferred Reporting Items for Systematic Reviews and Meta-Analyses
PCR-RFLP: Polymerase chain reaction-restriction fragment length polymorphism
HKSJ: Hartung-Knapp-Sidik-Jonkman
GRADE: Grading of Recommendations Assessment, Development and Evaluation
FDR: False discovery rate
MeSH: Medical Subject Headings
CNKI: China National Knowledge Infrastructure
SNP: Single nucleotide polymorphism
