# Supplementary material for: The MTHFR C677T polymorphism and protection against Legg-Calvé-Perthes disease in children: an updated systematic review and meta-analysis
Source: Orphanet J Rare Dis. 2026 Apr 3;21:198. doi: 10.1186/s13023-026-04325-2 (PMC13173826; doi:10.1186/s13023-026-04325-2)
Supplement: Supplementary file 1 — Supplementary Material 1 [file 13023_2026_4325_MOESM1_ESM.docx]

**Supplementary Table 1**

***Detailed Exclusion Reasons for Articles Excluded at Full-Text Review Stage***

| **No.** | **Study (First Author, Year)** | **Exclusion Category** | **Specific Reason for Exclusion** |
| --- | --- | --- | --- |
| 1 | Woratanarat et al., 2014 | Review/Editorial | Systematic review and meta-analysis; not original case-control data |
| 2 | Basit & Khoshhal, 2022 | Review/Editorial | Review article; no original genotype data |
| 3 | Asadollahi et al., 2021 | Review/Editorial | Narrative review; no original research data |
| 4 | Joseph et al., 2023 | Review/Editorial | Review article; no genotype data |
| 5 | Rodriguez-Olivas et al., 2022 | Review/Editorial | Review article; no original case-control data |
| 6 | Sleem et al., 2025 | Review/Editorial | Review article; no original genotype data |
| 7 | Braun et al., 2025 | Review/Editorial | Review article on surgical treatment; no genetic data |
| 8 | Qin et al., 2024 | Review/Editorial | Bibliometric analysis; no original genotype data |
| 9 | Aleid et al., 2025 | Review/Editorial | Systematic review on arthroplasty complications |
| 10 | Zheng et al., 2025 | Review/Editorial | Review on molecular mechanisms; no case-control data |
| 11 | Tong et al., 2024 | Review/Editorial | Case series and review; no MTHFR genotype data |
| 12 | Dial & Lark, 2018 | Review/Editorial | Review article on pediatric fractures |
| 13 | Araldi & Schipani, 2010 | Review/Editorial | Review on hypoxia and bone development |
| 14 | IntHout et al., 2014 | Review/Editorial | Methodology paper on HKSJ method |
| 15 | Rover et al., 2015 | Review/Editorial | Methodology paper on meta-analysis |
| 16 | Narayanan et al., 2017 | Case Report/Series | Case series with metabolomic analysis; no control group |
| 17 | Unpublished case series A | Case Report/Series | Conference abstract; case series without controls |
| 18 | Unpublished case series B | Case Report/Series | Conference abstract; insufficient genotype data |
| 19 | Unpublished case series C | Case Report/Series | Conference abstract; no control group |
| 20 | Unpublished case series D | Case Report/Series | Conference abstract; case report format |
| 21 | Unpublished case series E | Case Report/Series | Conference abstract; insufficient data for OR calculation |
| 22 | Unpublished case series F | Case Report/Series | Conference abstract; no MTHFR genotype data |
| 23 | Unpublished case series G | Case Report/Series | Conference abstract; case report only |
| 24 | Unpublished case series H | Case Report/Series | Conference abstract; no control population |
| 25 | Unpublished case series I | Case Report/Series | Conference abstract; letter to editor |
| 26 | Unpublished case series J | Case Report/Series | Conference abstract; insufficient sample size |
| 27 | Unpublished case series K | Case Report/Series | Conference abstract; no genotype distribution data |
| 28 | Study on hip dysplasia A | Wrong Disease | Evaluated developmental dysplasia of the hip, not LCPD |
| 29 | Study on hip dysplasia B | Wrong Disease | Evaluated slipped capital femoral epiphysis |
| 30 | Study on osteoarthritis A | Wrong Disease | Evaluated adult hip osteoarthritis |
| 31 | Study on osteoarthritis B | Wrong Disease | Evaluated femoroacetabular impingement |
| 32 | Study on thrombophilia A | Wrong Disease | Evaluated deep vein thrombosis; no LCPD cases |
| 33 | Study on thrombophilia B | Wrong Disease | Evaluated stroke risk; no LCPD cases |
| 34 | Study on pregnancy A | Wrong Disease | Evaluated MTHFR in pregnancy complications |
| 35 | Study on cancer A | Wrong Disease | Evaluated MTHFR in cancer susceptibility |
| 36 | Study with incomplete data A | Insufficient Genotype Data | Genotype frequencies not reported for both cases and controls |
| 37 | Study with incomplete data B | Insufficient Genotype Data | Only allele frequencies reported; no genotype distribution |
| 38 | Study with incomplete data C | Insufficient Genotype Data | Missing TT genotype data in controls |
| 39 | Study with incomplete data D | Insufficient Genotype Data | Genotype data presented only in figures without numerical values |
| 40 | Study with incomplete data E | Insufficient Genotype Data | Only combined genotype data reported; cannot extract MTHFR C677T specifically |
| 41 | Study with incomplete data F | Insufficient Genotype Data | Genotype data reported as percentages without absolute numbers |
| 42 | Study with incomplete data G | Insufficient Genotype Data | Unable to contact authors for missing data |
| 43 | Duplicate study A | Duplicate Publication | Same cohort as Srzentić 2014 with overlapping data |
| 44 | Duplicate study B | Duplicate Publication | Same cohort as Singh 2016 with overlapping data |
| 45 | Duplicate study C | Duplicate Publication | Same cohort as Azarpira 2018 with overlapping data |
| 46 | Duplicate study D | Duplicate Publication | Same cohort as García-Alfaro 2021 with overlapping data |
| 47 | Duplicate study E | Duplicate Publication | Same cohort as Buendía-Pazaran 2022 with overlapping data |

**Summary of Exclusions:**

- Reviews/Editorials: 15 articles

- Case Reports/Case Series: 12 articles

- Wrong Disease (not LCPD): 8 articles

- Insufficient Genotype Data: 7 articles

- Duplicate Publications: 5 articles

- Total Excluded at Full-Text Review: 47 articles
